# Active Ageing: An Empirical Approach to the WHO Model

**DOI:** 10.1155/2012/382972

**Published:** 2012-10-31

**Authors:** Constança Paúl, Oscar Ribeiro, Laetitia Teixeira

**Affiliations:** ^1^Research and Education Unit on Ageing, UnIFai, ICBAS, Institute of Biomedical Sciences Abel Salazar, Universidade do Porto, 4050-313 Porto, Portugal; ^2^School of Health Sciences, University of Aveiro, Campus Universitário de Santiago, 3810-193 Aveiro, Portugal; ^3^Instituto Superior de Serviço Social do Porto Cooperativa de Ensino Superior de Serviço Social, C.R.L., Avenue Dr. Manuel Teixeira Ruela, 370, 4460-362 Senhora da Hora, Portugal

## Abstract

*Background*. In the beginning of the 21st century, the world summit on population taking place in Madrid approved active ageing, WHO (2002) as the main objective of health and social policies for old people. Few studies have been done on the scientific validity of the construct. This study aims to validate the construct of active ageing and test empirically the WHO (2002) model of Active Ageing in a sample of community-dwelling seniors. *Methods*. 1322 old people living in the community were interviewed using an extensive assessment protocol to measure WHO's determinants of active ageing and performed an exploratory factor analysis followed by a confirmatory factor analyses. *Results*. We did not confirm the active ageing model, as most of the groups of determinants are either not independent or not significant. We got to a six-factor model (health, psychological component, cognitive performance, social relationships, biobehavioural component, and personality) explaining 54.6% of total variance. *Conclusion*. The present paper shows that there are objective as well as subjective variables contributing to active ageing and that psychological variables seem to give a very important contribute to the construct. The profile of active ageing is expected to vary between contexts and cultures and can be used to guide specific community and individually based interventions.

## 1. Introduction

The World Health Organization (WHO) defines active ageing as “… *the process of optimizing opportunities for health, participation, and security in order to enhance quality of life as people age*” [1]. The emergence of this concept back in the 1990s developed through the WHO and several other governmental and nongovernmental organization initiatives offers a policy framework that emphasizes the link between activity, health, independence, and ageing well. In being of unquestionable importance as a key policy concept, efforts to add some empirical evidence on its operative definition and criteria are still scarce. As a potential variation of other terms used interchangeably in the gerontological literature as positive and productive ageing, the interpretation of active ageing often focuses on the labour market participation anchored in an economic framework [2] or in a perspective strongly health oriented, though the WHO does take an multidimensional approach and a broad view of “health.” In fact, for many years WHO used to talk about healthy ageing, considering primary ageing without major pathologies, and only in the XXI century this concept was substituted by the more comprehensive concept of active ageing, considering not only health indicators but also psychological, social, and economic aspects, which are to be looked through communities' approaches within gender and cultural perspectives.

Notwithstanding the established importance of WHO's concept of active ageing as the leading global policy strategy in Europe [3], the scientific interest on its empirical dimension seems scarce at an international level. Based on a literature review using the key words “active ageing” and “WHO (2002)” on HighWire plus Medline, we found only 8 articles referring to the existence of the political framework proposed by WHO. In PsyInfo database, results were even scarcer with only two comments on Fernandez-Ballesteros' book on active ageing [4]. It seems that the document produced by WHO is more relevant in Europe than in the USA, with many countries introducing the model recommendations into their national health and social plans of action although, in general, it did not elicit many scientific discussion. In USA, researchers seem not to use the concept boosted by WHO in 2002 in their scientific papers and prefer to use the parallel concept of “successful ageing” as proposed by Rowe and Kahn [5, 6] when referring to ageing well or optimal ageing. As a matter of fact, for the concept “successful ageing” we found 3587 papers in the same data bases.

### 1.1. The Active Ageing Model

The concept of active ageing [1] is based on three pillars mentioned in the definition: participation, health, and security. The proposed model encompasses six groups of determinants, each one including several aspects: (1) health and social services (promoting health and preventing disease; health services; continuous care; mental health care); (2) behavioral (smoking; physical activity; food intake; oral health; alcohol; medication); (3) personal (biology and genetics and psychological factors); (4) physical environment (friendly environment; safety houses; falls; absence of pollution); (5) social (social support; violence and abuse; education); (6) economic (wage; social security; work), embedded in cultural and gender context, with recommendations for health policy for old people, to be implemented through national health plans all over the world, during the first decade of the XXI century.

According to the WHO document on active ageing [1], the key aspects of active ageing are (1) autonomy which is the perceived ability to control, cope with, and make personal decisions about how one lives on a day-to-day basis, according to one's own rules and preferences; (2) independence, the ability to perform functions related to daily living—that is the capacity of living independently in the community with no and/or little help from others; (3) quality of life that is “*an individual's perception of his or her position in life in the context of the culture and value system where they live, and in relation to their goals, expectations, standards, and concerns. It is a broad ranging concept, incorporating in a complex way the person's physical health, psychological state, level of independence, social relationships, personal beliefs, and relationship to salient features in the environment*.” [7]. As people age, their quality of life is largely determined by their ability to maintain autonomy and independence and (4) healthy life expectancy which is how long people can expect to live without disabilities. 

Active ageing appears as an outcome of different determinants that should allow us to identify particular profiles that are more at risk or, on the other hand, are more favorable to age actively.

### 1.2. Measuring Successful and Active Ageing

Recently, Pruchno et al. [8] wrote a paper on the early and contemporary characteristics of successful ageing. The authors stressed the proliferation of research on this topic over the past 50 years yet the inexistence of harmony on its definition and measure. The main point was to understand the influence of genetic and early experiences, as well as actual behaviors of individuals on ageing outcomes. Based on latent profile analyses, they concluded that successful ageing is a multidimensional construct that includes both objective and subjective characteristics, and that ageing outcomes can be modifiable by current behaviors. In another paper, Pruchno et al. [9] tested the two factor model of successful ageing by doing a confirmatory factor analyses. Factors were objective success (ample functional abilities, little or no pain, and few chronic diseases) and subjective success (perceptions of ageing successfully, ageing well, and overall evaluation of current state of one's life). They showed that age and gender were associated with objective but not subjective perceptions of successful ageing.

Previous discussion on the issue of objective versus subjective variables of successful ageing had stressed the idea that the proportion of people claiming ageing successfully is higher than the proportion of people classified as successful agers by objective indicators [10]. These authors found 92% of old people perceiving themselves as successful, although they were not free of disease or disability. The majority of subjects met the criteria for independent living, mastery, positive adaptation, life satisfaction, and active engagement, and only 15% met the criteria for absence of physical illness, and 28% reported no physical limitation. Successful ageing was not related to age, gender, ethnicity, marital status, education, and income which emphasize, in our view, the psychosocial variables of successful ageing over other characteristics of individuals.

In a different society (Taiwan), Lee et al. [11] confirmed a four-factor model of successful ageing. Again leisure activities appeared as a very relevant factor to the successful ageing process. Chaves et al. [12] studied the predictors of normal and successful ageing in urban old Brazilians and found 62% successful old people that fulfill the criteria of health and independence, differing from “normal” ones, namely, in the amount of leisure activities. In this same study, the number of living children appeared as a risk factor, whereas confidents and family income were protective factors of successful ageing. Authors discussed these findings considering that in developing countries as Brazil, contrarily to developed ones, socioeconomic status and social network seem to be more important than biological variables to predict successful ageing.

When examining the concept of ageing well in Europe and Latin America, Fernández-Ballesteros et al. [13, 14] found evidence of considerable consistency across countries, continents, and ages. The common thoughts toward ageing were that healthy ageing was the most important factor followed by independence (ability to manage oneself) and social implication which included positive affect. The ability to learn new things and the ability to work after retirement, as well as feeling able to influence others and staying involved with the world and people were considered less important. These results are quite similar to those of Bowling [15] that reported that over three-quarters of respondents were classified as ageing successfully, with self-perceived health status and quality of life as predictors of self-rated successful ageing. This author considers that the biomedical perspective of successful ageing needs balancing with a psychosocial one.

McLaughlin et al. [16] based on the Rowe and Kahn model [5, 6] had already estimated the prevalence of successful ageing on a national sample of older adults. The factors considered were disease and disability, cognitive and physical functioning, and social connections and productive activities. Results showed that only 11.9% individuals were ageing successfully every year, and that this percentage lowered in 25% between 1998 and 2004. The probability of being successful is lower for those with advanced age, male gender, and lower socioeconomic status. Based in this analysis, the authors considered that there is a need for modification in the concept of successful ageing for public health purposes. Depp and Jeste [17] made an extensive review on successful ageing studies and found in 28 selected studies that 26 of them included disability and very few psychosocial variables. The most frequent correlates of successful ageing were young age, no smoking, and absence of disability, arthritis, and diabetes. About 1/3 of individuals were ageing successfully, although the differences from study to study were large.

When explicitly exploring the concept of active ageing, Bowling [18] reported that a third of respondents rated themselves as ageing “very actively” and almost a half as “fairly actively.” The most common perceptions of active ageing were having/maintaining physical health and functioning (43%), leisure and social activities (34%), mental functioning and activity (18%), and social relationships and contacts (15%). The predictors of positive self-rated active ageing were optimum health and quality of life. More recently, Stenner et al. [19] reported the subjective aspects of active ageing by inquiring people about the meaning of the words “active ageing.” The authors showed that most people refer physical activity but also autonomy, interest in life, coping with challenges, and keeping up with the world. As mentioned, people mix physical, mental, and social factors and stressed *agentic capacities* and living by one's own norms. The authors criticized the deterministic view of the WHO model and emphasized the need for a “challenge and response” framework, a psychosocial approach to the conflict between facts and expectations, and the proactive attitude of people.

In overall, successful ageing, active ageing, and other related terms as positive ageing or ageing well are viewed as scientific concepts operationally portrayed by a broad set of biopsychosocial factors, assessed through objective and subjective indicators as well as being closely related to lay concepts reported cross-culturally by older persons [20]. Considering the heterogeneity of old people and the huge variety of individual trajectories, it is difficult, and probably ineffective, to define the core concept of successful ageing. A strict pattern of success excludes too much people all around the world, and an attempt to establish a standard for successful ageing, even a hypothetical biomedical objective standard, does not embrace the differences observed in old people (e.g., those with born or acquired incapacity). The concept of active ageing, although very difficult to measure, seems less deterministic, either as an outcome or as a process of achieving it. On the contrary, the well-known concept of successful ageing of Rowe and Kahn [5] looks more narrow and unrealistic, considering the very small amount of people (around 8.5%) that fulfill the criteria of ageing well [21].

In this paper, we explore the WHO's model of active ageing [1] that embraces positive outcomes of the ageing process. It is a challenge to examine the validity of the model and its empirical potential to foster quality of life in old people. Although we cannot really speak about “determinants of active ageing” as we cannot assert any causality without having a clear dependent variable and by doing a cross-sectional research, we intended to understand which and how the groups of variables are associated with active ageing. The main purpose of this research was to (i) built a protocol to assess WHO active ageing model and (ii) to verify which are the determinants that better explain active ageing.

## 2. Methods

### 2.1. Data Collection

This paper is part of an extensive Portuguese project on active ageing (DIA project) that includes a cross-sectional survey of adults aged 55+ years living in the community. For this study, subjects were recruited through announcements in local newspapers, local agencies (e.g., seniors clubs), and NGO's and using the snowball method by which participants indicate other persons with similar conditions. The study ran in different Portuguese regions, including the Madeira and Azores islands. The survey was conducted by trained interviewers, using a structured questionnaire format that entailed demographic, psychological, and social questions. A full description of the assessment protocol (P3A) can be found in Paúl et al. [22] and at http://www.projectodia.com. The interviews took place in local community facilities (e.g., parish hall) or at the participants' homes. Informed written consents were obtained from all the participants.

### 2.2. Sample Characteristics

The sample comprises 1322 persons aged 55–101 years old. The average age was 70.4 years (SD 8.7 years), and females comprised 71.1% (*n* = 939) of the sample. The majority of participants were married/partnered (*n* = 729, 55.7%), 400 (30.6%) were widowed, 114 (8.7%) were single, and 65 (5.0%) were divorced. As for the social network, 24.7% of the participants lived alone. Primary school education was reported by 55.3% of the respondents, 19.1% had never attended school, 17.8% had completed high-school, and 7.7% had higher education (trade qualification or university degree). Most participants (49.6%) had a monthly income equal or less than 386€ (by reference to the Portuguese Minimum National Wage in 2006). For the statistical analysis, as the distribution of missing values did not follow a pattern, participants with at least one missing response were eliminated, and the final sample contains 925 persons. The actual sample diverges from the national distribution of characteristics of old people [23], in the percentage of men and women in the sample, with a higher percentage of women in our sample than the existing in the Portuguese population 55+ years (71% versus 57% women) and the percentage of married individuals and widows (55.7% versus 71.1% married and 30.6% versus 20.1% widows). A special mention is to be made on the percentage of illiterate people in our study which is similar to the national figures: 19.1% versus 17% for people 15+ years.

### 2.3. Measures

The protocol measures the different groups of determinants of WHO's active ageing model and was elaborated considering an extensive literature review of most common instruments used in Gerontology and previously used the European Survey on Ageing Protocol [24] (Table 1). All instruments are adapted to Portuguese.

Along with socio demographic characteristics (gender, age, education, and income), we analyzed cognitive functioning as measured by the Portuguese version of the Minimental State Examination (MMSE) adapted to illiterate people and to people with very few years of education [25, 26]; social network was assessed with the Lubben Social Network Scale (LSNS) which comprises three subscales—family, friends, and confidants [27]; psychological distress was measured with General Health Questionnaire (GHQ-12) [28]; optimism was assessed with the Portuguese Version of the Life Orientation Test-Revised (LOT-R) [29, 30]; personality was evaluated with the NEO Personality Inventory [31] which comprises three subscales—neuroticism, extraversion, and openness to experience; happiness was assessed with a single question with four categories [32]; and environment domain of quality of life was measured with World Health Organization Quality of Life-BREF (WHOQOL-BREF) [7, 33] and Inventory of Life Satisfaction [34]. Biobehavioral measures, including pulmonary function and strength, were assessed using a standard “Mini Peak Flow Meter” (Datosprir Peak-10, Sibelmed) and with an electronic dynamometer (Grip-D, TAKEI Scientific Instruments Co., LTD), respectively. Finally, health and physical condition were evaluated by self-report indicators (determined by a standard health-rating item: “In general, how would you rate your health?”), illness (sum of self-reported health problems), sleep problems, subjective physical activity (determined by the item: “In general, how would you rate your physical condition?”), ADL, loneliness, vision, audition, smoking, and drinking. Details regarding variables and coding are shown in Table 2.

### 2.4. Exploratory Factor Analysis

The factor structure of P3A was examined by exploratory factor analysis, using principal-components extraction with varimax rotation. For the continuous variables, we used the quartiles in order to standardize the variables and use only categorical variables in the exploratory factor analysis. Exploratory factor analysis was conducted using SPSS 17.0 for Windows.

### 2.5. Confirmatory Factor Analysis

Confirmatory factor analysis was conducted to test the viability of a hypothesized structure that had been formulated from theoretical considerations and results of the exploratory factor analysis. Confirmatory factor analysis was conducted using AMOS 18 for Windows. Satisfaction scores for each dimension were obtained using factor score regressions generated from the confirmatory factor analysis as proportional weight to combine item scores. Our process of analysis started with the full factors and items, and then we used a nested models approach to test alternative nested structures to test fit improvement. In addition to theoretical and practical considerations, evaluation of fit of model was based on the following goodness of fit criteria, including normed chi-squared (*χ* ^2^/*df*), the comparative fit index (CFI), the goodness of fit index (GFI), the Akaike's information criteria (AIC), and the Browne-Cudeck criterion (BCC). CFI and GFI indices assume values in range from 0 to 1, with higher scores indicating better fit. Models with the lowest values of AIC are most likely to be good fits. We used the chi-square difference statistics to test the significance of the change in the chi-square test for each alternative model over the full model. Lastly, we examined the effect of age and gender on the final model estimating paths between age and gender and factors. Nonsignificant paths were removed, and the model was estimated over and over until only significant paths remained.

## 3. Results

### 3.1. Descriptive Analysis

Descriptive analysis (absolute and relative frequencies) was performed for all variables described in Table 2. When exploring the results, the variables “smoking” and “drinking” were excluded to the final analysis because distribution for this two variables were skewed, showing a pattern of responses in only one or two categories (e.g., no smokers; no heavy drinkers).

### 3.2. Exploratory Factor Analysis

The factor structure was examined by principal-components extraction with varimax rotation for the pooled sample (*n* = 925). The Bartlett sphericity test and the Kaiser-Meyer-Olkin (KMO) test were performed; the first revealed a 0.001 level of significance and a KMO value of 0.855, indicating that factor analysis seemed to be highly adjusted to this analysis. Six distinct factors, accordingly to the theoretical six determinants of the WHO model, were revealed (Table 3), explaining 54.6% of total variance. The item “hearing” was eliminated because it had a loading lower than 0.3 in all factors.

- Factor 1. Health component: this factor comprises five variables (subjective health, sleep problems, subjective physical condition, ADL, and illness) and explained 11.6% of total variance.
- Factor 2. Psychological component: six variables load heavily of this factor (psychological distress, happiness, optimism, neuroticism, quality of life—environment, and loneliness), which accounted for 11.2% of the total variance.
- Factor 3. Cognitive performance component: four questions have their highest loadings on this factor (cognitive impairment, vision, income, and education level) and explained 10.6% of total variance.
- Factor 4. Biological component: this factor comprises only two variables (peak flow and grip strength) and explained 7.7% of total variance.
- Factor 5. Social relationship component: three variables have their highest loadings on this factor (family, friends, and confidence), accounting for 6.9% of total variance.
- Factor 6. Personality component: the last factor contains only two variables (extraversion and openness to experience) and explained 6.6% of total variance.

**Figure 1:**
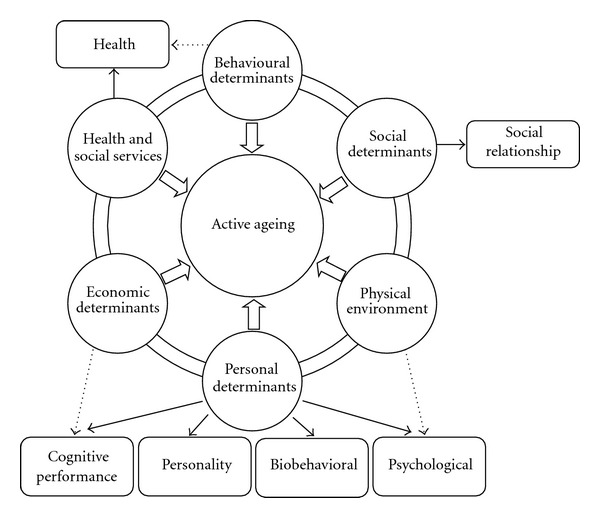
The WHO model and the empirically achieved model.

Comparing to the original model [1], our findings revealed a somewhat different one, depicted in Figure 1. *Health and social services determinants* merged with *behavior determinants* in a single component entitled “health” that includes functionality and life style. *Personal determinants* split into several components, namely, “psychological,” “cognitive performance,” “personality,” and “biobehavioral.” *Physical determinants* and *environment determinants* moved to the “psychological component” as a variable of perceived subjective well-being. *Economic determinants* migrated to the new component called “cognitive performance.” Only *social determinants *stayed as an independent factor that we renamed “social relationships.”

The achieved model shows that the “health component” is the major factor associated with active ageing and includes self-perception of health, the number of diagnosis, functionality (ADL and IADL), and life style. The second component was “psychological,” which is frequently forgotten in literature, with the exception of psychopathological indicators. In this study, psychological variables include both negative affect (psychological distress, loneliness, and neuroticism) and positive affect (happiness, quality of life—environment, and optimism). The “cognitive performance component” follows in weight showing the importance of wage, education, vision, and cognitive performance. The “biobehavioral component,” comprising respiratory capacity and grip strength clearly shows the importance of biological aspects during the ageing process. “social relationship”, including family, friends, and confidents, illustrates the relevance of social network for the quality of life of old people. Finally, the “personality component” was reduced to extraversion and openness to experience, as neuroticism merged with other psychological variables in the “psychological component.” The profile is quite homogeneous with factors loading between 11.6% and 6.6% and explaining a good amount of total variance (54.6%).

### 3.3. Confirmatory Factor Analysis

We analyzed the full six-factor model for the 22 variables by using the six item clusters derived from the exploratory factor analysis (presented in Table 3). From the results of this first full model that replicated the measurement structure derived from the original exploratory factor analysis, we proposed alternative models. We used a nested models approach to test alternatives to the full model (Model 1), elimination of item “sleep problems” (Model 2), elimination of item “vision” (Model 3), adding the following covarying error variances between “optimism” and “neuroticism” items and between “cognitive impairment” and “income” items (Model 4). However, these do not introduce any change in the final model. Fit statistics of the full model and subsequent models are presented in Table 4.

**Figure 2:**
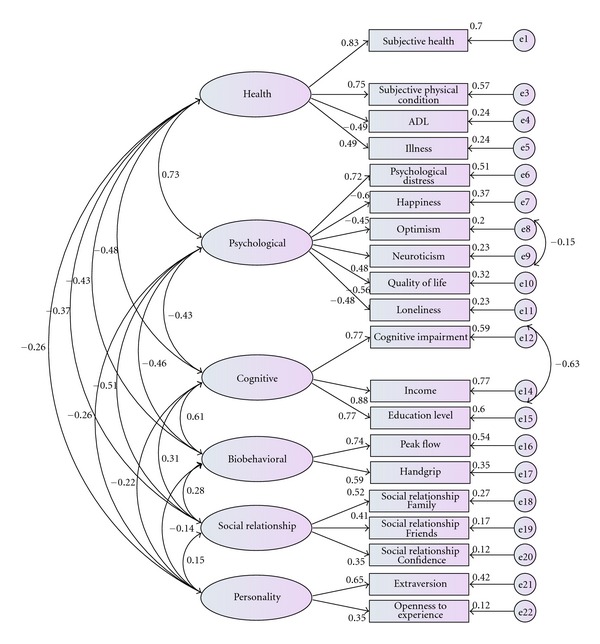
Factor structure model for P3A.

The confirmatory factor analyses structure describes adequately the 6 factors reinforcing the adequacy of the proposed model. The various indices of fit presented in Table 4 suggest that satisfaction structure can be adequately described by the 6 correlated factors which are graphically presented in Figure 2 (Model 4). Latent constructs (active ageing components) are shown as ellipses, and questionnaire items measuring these latent constructs are represented as rectangles.

Finally, testing the effects of age and gender, only the paths between gender and the “cognition component” and gender and the “Biobehavioral component” were significant (*P* < 0.05, for both). Nonsignificant paths were removed, and the final model revealed that the model fit the data very well (*χ* ^2^ = 624.19, *df* = 171, *P* < 0.001, CFI = 0.906, GFI = 0.941). Women had higher levels of “Cognitive performance component” and lower levels of “Biobehavioral component.”

## 4. Discussion

When we look at the WHO model we can see that apart from the *social determinant* all the others endured a rearrangement that lead to six factors not similar to the original ones. However, “active ageing” remained a complex construct, where health and psychological adaptation play the major role. Many of the determinants proved to be entwined, reflecting the transaction between individual and environmental factors in shaping adaptation to the ageing process.

The *economic determinants* as well as the *physical environment *and *health and social services* relevance were found to be associated with personal needs, resources, and outcomes and do not configure independent factors. According to our findings, people seem to perceive and assess reality concerning social and personal conditions through the glasses of their own values and needs, adding to their real circumstances a self-perceived valuation of what they are experiencing. Globally, we can say that subjective and objective health and functionality constitute the main component of active ageing which goes in line with Pruchno et al. [8, 9] findings; the psychological component, be it positive characteristics of individuals (e.g., happiness, optimism) or pathological ones (e.g., psychological distress, neuroticism), is the second most relevant factor, reinforcing the idea of positive affect associated with less mortality and longevity (e.g., [35]); cognition appearing close to vision supports Baltes and Mayer's [36] findings on the importance of senses in cognition and in the overall optimal ageing. Income and education levels that contribute for this factor show, on one hand, the importance of cognition in the process of ageing and, on the other hand, a close association between income, access to education, and cognitive performance. Biological variables proved to be very sensitive to gender and age as expected (e.g., peak flow and grip strength), and contributing independently to active ageing; social relationship including family, friends, and confidents networks supports Bowling's [15] findings on the importance of social networks to successful ageing; finally, personality seems to introduce a factor of more or less adaptability to the challenges of ageing.

This achieved six-factor model reveals the major contributions the active ageing constructs and goes beyond the successful ageing model that establishes a strict pattern of success by considering that different profiles of old people in different contexts may be classified as active with areas in debt being compensated by more advantaged ones. The relative load of each factor will presumably change in diverse contexts or groups of people, emphasizing the need for different intervention programs to foster quality of life allocating diverse life trajectories, and where, for instance, high income can compensate smaller social networks or optimistic disposition can compensate disability to balance positively the process of ageing. Furthermore, rather than health problems that most of old people have (and/or expected to have in some extent) and some functional limitations, the difference between old people ageing actively or not may vary with the psychological characteristics and status that enable them to cope with ageing related declines, look forward, and keep committed to life. By keeping active in the broader sense of the concept, old people seem to overcome difficulties and keep highly motivated to participate in the social world and engage in healthy behaviors which raise quality of life during the ageing process. As stated recently here, a psychological approach to successful ageing is to have a crucial role in predicting future quality of live in older adults, namely, by maximizing one's self-efficacy and resilience [37].

The WHO active ageing model [1] based on 6 determinants was not empirically validated in its structure for the sample here considered. Some groups of determinants were found to be deeply intertwined. The proposed model requires further developments, namely, by studying psychological mechanisms that might be related to the ability to cope with ageing, and particularly among the very old. Culture-based approaches are also to be considered in future studies.

This study has two main limitations. The first one regards to the exclusive use of self-rated measures that may had led to an overall “perceived reality” whilst some of the active ageing determinants are to be more objective (e.g., actual presence of social and health services), although Portugal has a NHS with universal and free access and a reasonable coverage of services for the elderly (nursing homes and day centres and a not so extensive service of home care). On this aspect, it is worthwhile mentioning that most of the municipalities have conventional services for old people and that self-report of availability and satisfaction of community health and social services is thought to better reflect the reality and the experience of the present cohort of old people. Moreover, the use of mostly self-reported measures except for cognitive performance and biological parameters, although missing clinical diagnosis and objective environmental variables, constitutes a reliable overview of old people perspective of their own condition and that of the context in which they live. Both these aspects must be considered when interpreting our findings and when conducting further research. The second main limitation has to do with the sampling process (e.g., using announcements in newspapers, senior clubs) which may have resulted in a selection towards the most active older adults. We consider that further studies should comprise different sample selection procedures and a wider coverage of older people towards a more representative overview of the Portuguese population.

The challenge of active ageing is health and independent functioning, whereas psychological variables appear to be highly relevant determining the individual adaptation to the ageing process. In this sense, interventions are to consider the prevention of health problems from adulthood and the increasing of psychological resilience, avoiding loneliness or increasing happiness and subjective wellbeing. Other social and political variables demand different kinds of intervention at a community-based level, namely, rising income and carefully planning the retirement process and pensions regimens.

## Figures and Tables

**Table 1:** Instruments used for each of the WHO's active ageing model determinants.

Determinants	WHO (2002) contents	Assessment protocol “P3A”	
Personal factors	Biology and genetics psychological factors	Psychological distress	GHQ-12 [28]
		Happiness	QBE/F [32]
		Cognitive functioning	MMSE [25]
		Personality	NEO (Costa and McCrae, 1992 [31])
		Optimism	LOT-R [30]
		Loneliness	Loneliness scale (Paúl et al., 2008 [22])

Behavior determinants	Smoking Physical activity Food intake Oral health Alcohol Medication	Pulmonary function	Peak flow
		Strength	Hand grip
		Subjective health	Health and life styles questionnaire (ESAP, Fernández-Ballesteros et al., 2004 [24])
		Illness	
		Sleep problems	
		Subj. physical activity	
		Vision	
		Audition	
		Smoking	
		Drinking	
		ADL and IADL	

Determinants of social environment	Social support	Social network	Lubben scale of social support (Lubben, 1988) [27]
	Violence and abuse		
	Education	Education	Sociodemographic questionnaire

Determinants of health and social services	Health and disease	Life satisfaction	Inventory of life satisfaction (Fonseca et al., 2011 [34])
	Health services		
	Continuous care		
	Mental health care		

Determinants of physical environment	Friendly environment	Environment domain of quality of life	WHOQOL Brief—physical environment subscale (Harper et al.,1998 [7], Canavarro et al., 2010 [33])
	Safety houses		
	Falls		
	Absence of pollution		

Economic determinants	Wage		Socioeconomic status (National Institute of Statistics)
	Social security	Income	
	Work		

**Table 2:** Definition of variables.

Variable	Coding
Subjective health	1 = very good; 2 = good; 3 = reasonable; 4 = poor; 5 = very poor
Sleep problems	0 = no; 1 = yes
Subjective physical activity	1 = very good; 2 = good; 3 = reasonable; 4 = poor; 5 = very poor
ADL	0 = with difficulties; 1 = without difficulties
Illness	0 = none;1 = 1 illness; 2 = 2 illness; 3 = 3 illness; 4 = 4 or more illness
Psychological distress^a^	1 = <9; 2 = [9,12[; 3 = [12,16[; 4 = ≥16
Happiness	1 = nothing; 2 = 2; 3 = 3; 4 = very
Optimism^a^	1 = <11; 2 = [11,13[; 3 = [13,15[; 4 = ≥15
Quality of life^a^	1 = <24; 2 = [24,26[; 3 = [26,29[; 4 = ≥29
Loneliness	0 = yes; 1 = no
Cognitive impairment^a^	1 = <25; 2 = [25,28[; 3 = [28,30[; 4 = ≥30
Vision	1 = no specs and very poor/poor vision; 2 = no specs and acceptable vision; 3 = no specs and good/very good vision; 4 = specs and very poor/poor vision; 5 = specs and acceptable vision; 6 = specs and good/very good vision
Audition	1 = no device use and very good/good audition; 2 = no device use and acceptable audition/3 = no device use and poor/very poor audition; 4 = use device
Smoking	1 = no; 2 = ex-smoker; 3 = yes
Drinking	1 = never; 2 = special occasions; 3 = occasionally; 4 = regularly
Income^b^	1 = ≤386 €; 2 = 386 €–772 €; 3 = 772 €–1158 €; 4 = >1158 €
Education level	1 = no formal; 2 = primary; 3 = 5–8 years; 4 = 9–12 years; 5 = university
Peak flow^a^	1 = <180; 2 = [180,250[; 3 = [250,340[; 4 = ≥340
Grip strength^a^	1 = <18.3; 2 = [18.3,22.9[; 3 = [22.9,29.0[; 4 = ≥29.0
Family^a^	1 = <9; 2 = [9,11[; 3 = [11,13[; 4 = ≥13
Friends^a^	1 = <5; 2 = [5,8[; 3 = [8,10[; 4 = ≥10
Confidents^a^	1 = <4; 2 = [4,7[; 3 = [7,9[; 4 = ≥9
Neuroticism^a^	1 = <30; 2 = [30,34[; 3 = [34,37[; 4 = ≥37
Extraversion^a^	1 = <39; 2 = [39,41[; 3 = [41,44[; 4 = ≥44
Openness to experience^a^	1 = <35; 2 = [35,37[; 3 = [37,40[; 4 = ≥40

^a^Quartiles; ^b^by reference to the Portuguese Minimum National Wage in 2006.

**Table 3:** Factor structure of P3A—exploratory factor analysis.

Questions	Factors					
	1	2	3	4	5	6
Subjective health	**0.652**	−0.298	−0.312	−0.071	−0.131	−0.104
Sleep problems	**0.620**	−0.154	0.152	0.133	−0.114	0.260
Subjective physical condition	**0.670**	−0.218	−0.250	−0.061	−0.104	−0.223
ADL	**−0.563**	0.052	0.262	0.160	−0.103	0.139
Illness	**0.673**	−0.067	0.004	−0.241	0.009	0.035
Psychological distress	0.437	**−0.586**	−0.101	−0.084	−0.112	−0.005
Happiness	−0.265	**0.540**	0.105	−0.085	0.260	0.213
Optimism	−0.050	**0.683**	−0.035	0.039	0.065	0.068
Neuroticism	0.096	**−0.695**	−0.114	−0.163	0.171	0.108
Quality of life—environment	−0.076	**0.616**	0.286	0.075	0.051	0.132
Loneliness	−0.149	**0.492**	−0.011	0.126	0.351	−0.084
Cognitive impairment	−0.096	0.180	**0.594**	0.396	0.103	−0.146
Vision	−0.100	−0.001	**0.592**	−0.211	0.056	0.242
Income	−0.162	0.135	**0.699**	0.261	0.126	−0.198
Education level	−0.098	0.133	**0.807**	0.204	0.034	−0.199
Peak flow	−0.044	0.157	0.295	**0.700**	0.056	−0.051
Grip strength	−0.266	0.098	0.042	**0.782**	0.060	0.058
Social relations—family	−0.028	0.109	−0.006	0.112	**0.727**	−0.063
Social relations—friends	−0.131	0.074	0.130	0.078	**0.400**	0.269
Social relations—confidence	0.024	0.013	0.104	−0.065	**0.700**	0.011
Extraversion	−0.196	0.106	−0.027	−0.199	0.055	**0.655**
Openness to experience	0.123	0.014	−0.190	0.143	−0.016	**0.734**

% of variance explained	11.6	11.2	10.6	7.7	6.9	6.6

**Table 4:** Goodness-of-fit statistics for confirmatory factor analysis models of P3A.

Model	*χ* ^2^	df	*χ* ^2^/df	CFI	GFI	*χ*_dif_^2^	AIC	BCC
1	701.342	194	3.615	0.891	0.936	—	819.342	822.354
2	562.046	172	3.268	0.913	0.946	139.30	680.046	682.924
3	557.039	155	3.594	0.908	0.944	5.01	667.039	669.597
4	489.170	153	3.197	0.923	0.950	67.87	603.170	605.822
